# Marine Myxobacteria: A Few Good Halophiles

**DOI:** 10.3390/md16060209

**Published:** 2018-06-14

**Authors:** Hanan Albataineh, D. Cole Stevens

**Affiliations:** Department of BioMolecular Sciences, School of Pharmacy, University of Mississippi, University, MS 38677, USA; haalbata@go.olemiss.edu

**Keywords:** marine myxobacteria, *Haliangium ochraceum*, *Enhygromyxa salina*, *Plesiocystis pacifica*, *Haliangium tepidum*, *Enhygromyxa niigataensis*, *Pseudenhygromyxa salsuginis*, *Paraliomyxa miuraensis*

## Abstract

Currently considered an excellent candidate source of novel chemical diversity, the existence of marine myxobacteria was in question less than 20 years ago. This review aims to serve as a roll call for marine myxobacteria and to summarize their unique features when compared to better-known terrestrial myxobacteria. Characteristics for discrimination between obligate halophilic, marine myxobacteria and halotolerant, terrestrial myxobacteria are discussed. The review concludes by highlighting the need for continued discovery and exploration of marine myxobacteria as producers of novel natural products.

## 1. Introduction

In a myxobacterial ecology review published in 1999, Hans Reichenbach asked, “Are there marine myxobacteria?” [1]. Reichenbach’s succeeding paragraph provides insight into the uncertainty surrounding marine myxobacteria prior to the routine practice of molecular taxonomy [1]. Two of the major factors contributing to this obscurity were the incorrect assignment of marine *Bacteroidetes* as lower order myxobacteria or ‘Myxobacteria imperfecta’ and the ubiquitous distribution and reported isolations of myxobacteria, with varying halotolerances among interfacial environments, such as sediments from beaches and shores [1,2,3,4,5,6,7,8,9]. While the 16s rRNA sequence analysis has mostly addressed the former, determining that what were considered lower-order marine myxobacteria, were instead *Bacteroidetes* from the genera *Cytophaga* and *Flavobacterium*, the latter continues to obfuscate the distinction between marine and terrestrial myxobacteria [10,11,12]. Literature concerning the halotolerant myxobacterium *Myxococcus fulvus* HW-1 (ATCC BAA-855) typifies this issue [13,14]. While *M. fulvus* HW-1 has been reported to tolerate a salinity as high as 3% and is listed in the World Register of Marine Species (WoRMS), the strain displays attenuated morphologies and social behaviors that are typical of myxobacteria, such as a fruiting body formation on agar media with low concentrations of seawater or salts [13,15]. This review aims to clarify such confusion by highlighting the unique characteristics of halophilic myxobacteria when compared to better-known halotolerant soil myxobacteria, and to encourage further discovery and investigation of marine myxobacteria as a source of structurally unique secondary metabolites.

## 2. Characteristics Unique to Marine Myxobacteria

The recent retrospective analysis of natural product discovery trends reported by Pye et al. concluded that marine organisms are, at least upon discovery, productive sources of novel chemical diversity [16]. Considering this observation, combined with the abundance of biologically active myxobacterial metabolites, we anticipate that an investigation of marine myxobacteria, as producers of secondary metabolites with unique molecular scaffolds and activities, will become a priority for drug discovery efforts [17,18,19]. While limited by the scarcity of cultivable marine myxobacteria, the following characteristics are meant to differentiate known terrestrial, halotolerant myxobacteria from marine, halophilic myxobacteria. These characteristics are centered on halophilicity, multicellular behaviors that have been observed at saline cultivation conditions, phylogeny, and ecology.

Halophilic bacteria rely on the cellular accumulation of organic osmolytes to prevent dehydration in osmotic environments, such as seawater [20,21]. Recently, differing strategies for osmolyte accumulation were reported for *Enhygromyxa salina* SWB007 and *Pleciocystis pacifica* SIR-1^T^ [22]. While *P. pacifica* accumulates amino acids to offset osmotic stress, *E. salina* produces the ubiquitous osmolytes betaine and hydroxyectoine [22]. Interestingly, all of the sequenced myxobacterial halophiles possess a betaine/carnitine/choline transporter (BCCT) or BetT homolog, while the sequenced terrestrial myxobacteria do not, regardless of the reported halotolerances (Table 1). The gene loci that encode solute biosynthetic pathways, such as the confirmed ectoine/hydroxyectoine cluster from *E. salina* SWB007, cannot currently be considered as critical aspects of marine myxobacteria, as *P. pacifica* SIR-1^T^ has no such pathway and instead relies on the accumulation of glutamate and glycine to prevent osmotic stress [22]. Excluding *M. fulvus* HW-1, the only cultivable myxobacteria found to be obligate halophiles have been associated with the ‘marine’ moniker.

While among the order of the *Myxococcales* obligate halophilicity is wholly unique to marine myxobacteria, all myxobacteria participate in unique multicellular behaviors, such as fruiting body formation, social swarming, and organized predation [23,24,25]. Salinity impedes fruiting body formation of the vast majority of terrestrial and halotolerant myxobacteria [13]. Instead, halotolerant myxobacteria adopt a less complicated, unicellular growth strategy that is independent of cell density, when grown at salinities reflective of seawater conditions [13]. While this strategy provides the resilience required for halotolerant myxobacteria to survive in sandy beaches and shoreline soils, it impedes their ability to thrive in seawater. An outlier, halotolerant *Pseudenhygromyxa salsuginis* SYR-2^T^, designated a ‘brackish water myxobacterium’, displays fruiting bodies similar to *E. salina* SHK-1^T^ and *P. pacifica* SIR-1^T^ at various levels of salinity [26,27,28]. Synchronized motility, referred to as swarming or gliding, is a defining feature of myxobacteria, has been observed for all marine myxobacteria as well as halotolerant myxobacteria, such as *M. fulvus* HW-1, when grown at varying salinities [12,25,26,27,28,29,30]. Halophilic myxobacteria swarm in radial patterns with varying amounts of etching occurring on the surface of agar medias [12,26,27,28,29,30]. Typical of terrestrial myxobacteria, marine myxobacteria do not form radial veins or distinct waves when swarming [12,26,27,28,29,30]. Instead, marine myxobacteria aggregate at peripheral bands along the outer circumference of swarms [12,26,27,28,29,30]. Unlike the predation efforts of the model myxobacterium *Myxococcus xanthus*, organized predation strategies of marine myxobacteria have yet to be explored in detail. While all halophilic myxobacteria discovered to date are capable of lysing a variety of Gram-negative bacteria, only *H. ochraceum, H. tepidum*, and *Enhygromyxa niigatensis* are able to lyse *Saccharomyces cerevisiae* cells [26,27,28,29,30].

**Figure 1:**
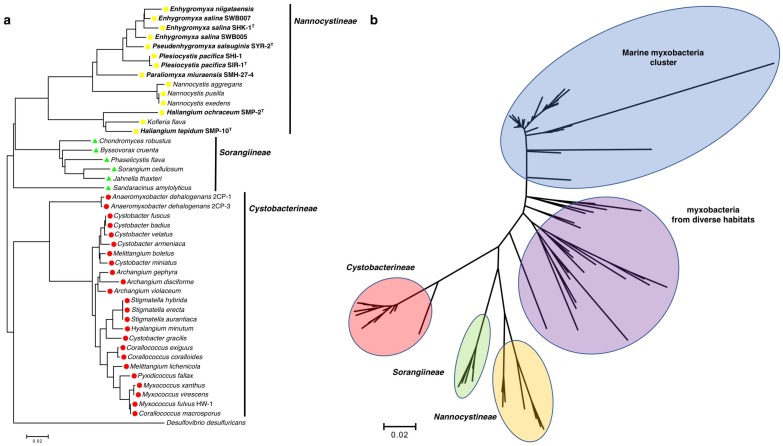
(**a**) Phylogenetic tree of cultivable *Myxococcales* with the discussed myxobacteria bolded; (**b**) Phylogenetic tree of myxobacteria, that includes the MMC and other myxobacteria from [31]. Using MEGA7, 16s rRNA sequences were aligned with ClustalW using general settings (gap opening penalty 15.0; IUB DNA weight matrix), and phylogenetic trees was generated using the neighbor-joining method [33].

The suborder *Nannocystineae* exclusively consists of halotolerant and halophilic myxobacteria, including all of the discovered cultivable halophilic myxobacteria, with the only potential outlier being the lesser studied, terrestrial *Kofleria flava* (Figure 1a). Yet again exceptional, *M. fulvus* HW-1 is the only purported marine myxobacterium to instead belong to the suborder *Cystobacterineae* (Figure 1a). However, Brinkhoff et al. recently reported a distinct cluster exclusively comprised of marine myxobacteria (Figure 1b) [31]. Aptly designated as the marine myxobacteria cluster (MMC), associated marine myxobacteria were observed primarily from sediment samples and water column samples near the sediment surface worldwide, at salinities ranging from brackish to marine [31]. Interestingly, the myxobacteria distinctly clustered between the MMC and the defined suborders *Nannocystineae*, *Sorangiineae*, and *Cystobacterineae* were from a variety of diverse habitats including volcanic sediment, a wastewater treatment plant, a glacier, a uranium mining waste pile, a microbial biofilm, a hypersaline microbial mat, and various marine sediments [31]. The prevalence of halotolerant and halophilic myxobacteria within the suborder *Nannocystineae*, the observation of clustered myxobacteria from fluid habitats, and the exclusivity of the MMC provide a unique phylogenetic landscape within the order *Myxococcales*, where physiological adaptability to environmental volatility seems apparent [13,25,31,32]. The observation of phylogeographic separation of marine and terrestrial myxobacteria, reported by Jiang et al., supports this conspicuous delineation [32]. While only recently observed, the MMC and the assumed capacity of the myxobacteria within to produce secondary metabolites exemplifies the need for continued efforts focused on the isolation and cultivation of marine myxobacteria.

## 3. *Haliangium ochraceum*

**Figure 2:**
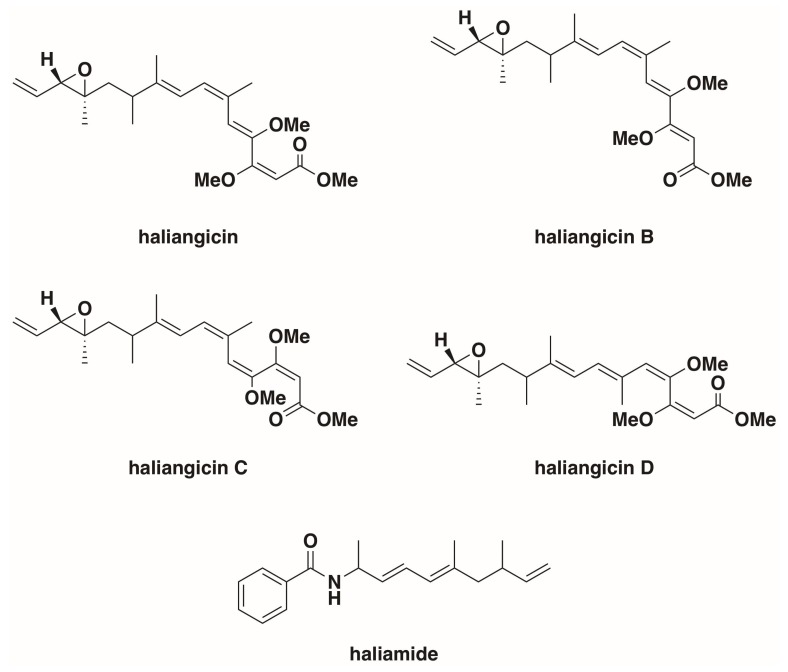
*Haliangium ochraceum* secondary metabolites [35,36,37,38]. Of note, *cis* isomers about the epoxide moiety have also been reported for all of the haliangicins [37].

Originally isolated from a seaweed sample collected from a beach on the Miura Peninsula of Japan and reported as *Nannocystis* sp. strain SMP-2 in 1998 by Iizuka et al., *H. ochraceum*, along with what would become *Plesiocystis pacifica*, was one of the first halophilic marine myxobacteria, which were confirmed by 16s rRNA sequencing to be a member of the myxobacterial suborder *Nannocystineae* [12,29]. The genome for type strain *H. ochraceum* SMP-2^T^ (DSM 14365^T^) was published in 2010 (NC_013440.1) [34]. Originally cultivated at 30–34 °C on modified VY/2 agar media (Baker’s yeast 5 g L^−1^, cyanocobalamin 0.5 mg L^−1^, agar 15 g L^−1^) supplemented with sea water, *H. ochraceum* was observed to grow at NaCl concentrations of 0.2–5% (*w*/*v*), with optimal growth between 2–3% [12,29]. Fudou et al. reported the first discovery of a secondary metabolite from marine myxobacteria, haliangicin, a polyketide with antifungal activity isolated from *Haliangium luteum* in 2001 [35,36]. Fudou et al. later reclassified *Haliangium luteum* as *H. ochraceum* [29]. *H. ochraceum* has since been reported to produce a variety of haliagicin congeners, as well as the hybrid polyketide-nonribosomal peptide haliamide (Figure 2), via a type-I polyketide synthase pathway and a hybrid polyketide-nonribosomal biosynthetic pathway respectively [37,38,39,40]. The features of *H. ochraceum*, denoted as differentiated from soil myxobacteria, include obligate halophilicity, palmitic acid as a principle fatty acid, and presence of anteiso-branched fatty acids [29]. Fruiting body formation has been reported from both solid and liquid cultures of *H. ochraceum*, regardless of salinity [13,29]. *H. ochraceum* swarms form slightly sunken radial bands within agar, generating a tough slime film [29]. Associated with the *Haliangiaceae* clade of the suborder *Nannocystineae*, *H. ochraceum* shows a higher 16S rRNA sequence similarity to terrestrial myxobacteria than other halophilic marine myxobacteria [36,37]. As previously mentioned, *H. ochraceum* is capable of lysing both Gram-negative bacteria, specifically *Escherichia coli* and *Micrococcus luteus*, but also *S. cerevisiae* [29].

## 4. *Enhygromyxa salina*

**Figure 3:**
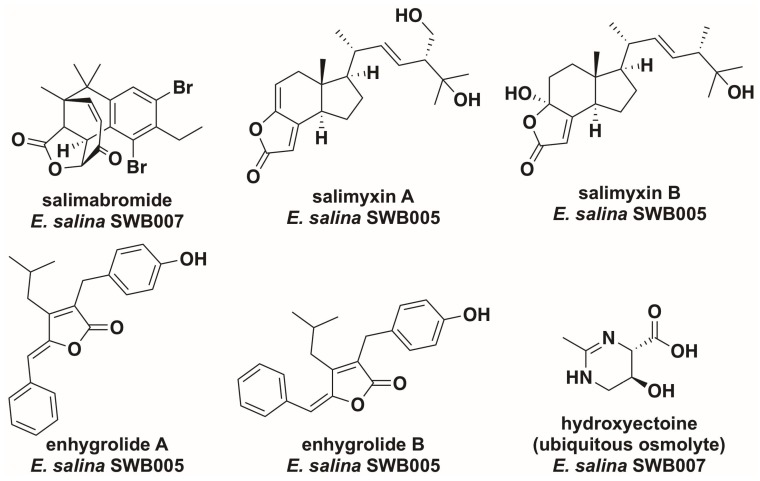
*Enhygromyxa salina* secondary metabolites and hydroxyectoine [20,43,44].

Also discovered by Iizuka et al., *E. salina* was initially isolated from a lagoon shore on the north coast of Hokkaido, Japan [27]. Three strains of *E. salina* have been sequenced, including the type strain *E. salina* SMK-1^T^ (DSM 15217^T^) and strains SWB005 and SWB007 [30,41]. Belonging to the *Plesiocystis/Enhygromyxa* clade of the suborder *Nannocystineae*, numerous unique strains of *E. salina* have been reported [27,30,42]. *E. salina* produces fruiting bodies varying from white to orange when grown on VY/2 supplemented with salt water [27]. An obligate halophile, *E. salina* tolerates NaCl concentrations of 0.1–4.0% (*w*/*v*) with an optimal range of 1.0–2.0% NaCl [27,30]. However, the numerous strains of *E. salina* all demonstrate varying ranges of salt tolerance with minimum concentrations as high as 1% NaCl (*w*/*v*) and maximum concentrations of 7% NaCl [27,30]. *E. salina* swarms form circular patterns, leaving deeply etched craters within agar surfaces [27]. While able to survive on media with yeast as the sole nitrogen source, *E. salina* is only capable of lysing Gram-negative bacteria and is unable to lyse *S. cereviseae* cells [27]. Numerous secondary metabolites have been discovered from a variety of *E. salina* strains (Figure 3); the activities and biosynthetic assembly of these metabolites have been well reviewed elsewhere [17,18,19,43,44]. Of note, comparative antiSMASH analysis of the three sequenced strains of *E. salina* suggests strain SWB005 to be the only sequenced strain of *E. salina* without a predicted trans-AT polyketide synthase, and strain SWB007 to be the only strain with an identified thiopeptide biosynthetic pathway [45]. Interestingly, only *E. salina* seems to produce the osmolyte hydroxyectoine, as no other EctD homologue is apparent in the genomes of other sequenced marine myxobacteria (Table 1) [22].

## 5. *Plesiocystis pacifica*

Discovered, yet again, by Iizuka et al., *P. pacifica*, originally identified as *Nannocystis* sp. SHI-1, was isolated from a beach on Iriomote-jima Island, Japan in 1997 [12,28]. There are currently two reported strains, the type strain *P. pacifica* SIR-1^T^ (DSM 14875^T^) and SHI-1 (DSM 14876) [28]. Both strains produce pinkish-orange to brownish-orange fruiting bodies when grown on VY/2 that is supplemented with salt water, and require NaCl concentrations of 1% (*w*/*v*) for growth with optimum salinities of 2.0–3.0% [28]. *P. pacifica* forms radial bands at the perimeter of its swarms, leaving cloudy etches in agar surfaces [28]. Similar to the other members of the *Plesiocystis/Enhygromyxa* clade of the suborder *Nannocystineae, P. pacifica* lyses Gram-negative bacteria and is unable to lyse *S. cerevisiae* [28]. While no secondary metabolites have been reported from either strain, an antiSMASH analysis on the draft genome of *P. pacifica* SIR-1^T^ (GCA_000170895.1) revealed numerous secondary metabolite biosynthetic pathways [18,46]. This analysis portrays *P. pacifica* as an excellent candidate for future natural product discovery efforts. Instead, the haloalkane dehalogenase DppA from *P. pacifica* SIR-1^T^ has garnered interest as a potential biocatalyst for bioremediation of aromatic pollutants [47,48]. DppA shows unique specificities towards brominated α,β-haloalkanes, with no activity observed towards the chlorinated substrates [48]. Only briefly referenced in a PCR survey of polyketide synthase genes from various myxobacteria, genomic DNA from *Plesiocystis* sp. strain SIS-2 was found to contain several polyketide synthases [46]. However, whether *Plesiocystis* sp. strain SIS-2 is a third strain of *P. pacifica* or a unique member of the genera *Plesiocystis* remains unclear.

## 6. *Haliangium tepidum*

The lesser investigated of the marine members of the *Haliangiaceae* clade of the suborder *Nannocystineae*, *Haliangium tepidum* SMP-10^T^ (DSM 14436^T^) was first reported by Fudou et al. [29]. Found to be an obligate halophile, *H. tepidum* grows and produces fruiting bodies at salinities ranging from 0.5–6.0% NaCl (*w*/*v*) [29]. *H. tepidum* is able to lyse both Gram-negative bacteria and *S. cereviseae*, and swarms in radial patterns leaving slime sheets slightly etched into agar surfaces [29]. As the species designation suggests, *H. tepidum* grows at moderately warm temperatures when compared to other marine myxobacteria with an optimal temperature range of 37–40 °C [29]. While no genome sequence data or discovered natural products have been reported, a PCR survey of myxobacterial genomic DNA found an abundance of polyketide synthases within the genome of *H. tepidum* [46].

## 7. Potential Marine Myxobacteria

**Figure 4:**
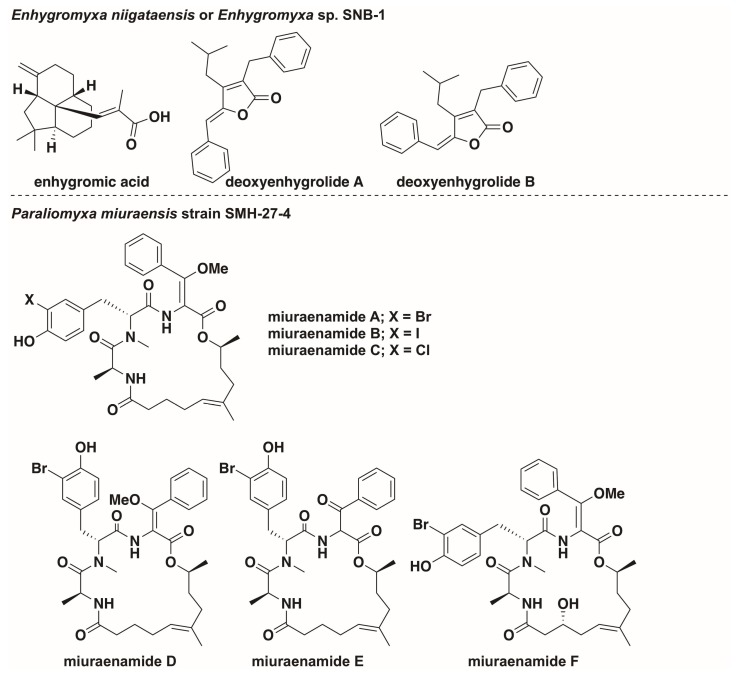
Secondary metabolites from potential marine myxobacteria [49,50,51].

While the six previously discussed marine myxobacteria are the best characterized to date, there have been several recently reported potential marine myxobacteria. Tomura et al. discovered three new myxobacterial natural products that were produced by *Enhygromyxa niigataensis* or *Enhygromyxa* sp. SNB-1 (Figure 4) [49]. With a 97% similarity to the 16S rRNA sequence of *E. salina* SWB004, *E. niigataensis* SNB-1 was determined to be a new species within the genera *Enhygromyxa*. Although the phylogenetic position of *E. niigataensis* would suggest that it is indeed a marine myxobacterium, the halotolerance levels and morphological features of the strain are currently unreported [49]. Originally isolated from a marsh bank in Shikoku, Japan, *Pseudenhygromyxa salsuginis* SYR-2T (DSM 21377T) develops fruiting bodies at NaCl concentrations of up to 2.5% (*w*/*v*), and forms slightly sunken, radial swarms [26]. However, *P. salsuginis* is not an obligate halophile [26]. Optimal growth conditions for *P. salsuginis* were determined to be somewhat saline between 0.2–1.0% NaCl [26]. The species identifier for *P. salsuginis*, translated as “of brackish water”, aptly summarizes this observation. The obligate halophile *Paraliomyxa miuraensis* SMH-27-4 produces a variety of halogenated hybrid polyketide-nonribosomal peptide metabolites (Figure 4) [50]. Although its halophilic nature strongly suggests the strain to be a marine myxobacterium, the morphological features for *P. miuraensis* at saline cultivation conditions have not been reported.

## 8. Conclusions

Ubiquitous to marine environments worldwide, cultivable marine myxobacteria remain a relatively underexplored resource [31]. Ideally, this roll call for known marine myxobacteria and corresponding descriptions will provide clarity to the early literature surrounding halophilic and halotolerant myxobacteria, as well as encourage the continued discovery of new marine myxobacteria. The recent expansion of the order *Myxococcales*, with the addition of myxobacteria associated with the MMC, suggests that the vast majority of marine myxobacteria have yet to be discovered [31]. Although the limited number seems to suggest a scarcity, it should be recognized that the covered marine myxobacteria have been discovered thanks to the tremendous efforts of only a few research groups. This dearth of cultivable marine myxobacteria has not, however, limited the chemical diversity of their cognate reported natural products. To date, natural product classes discovered from marine and potential marine myxobacteria include polyketides, hybrid polyketide-nonribosomal peptides, degraded sterols, diterpenes, cyclic depsipeptides, and γ-alkylidenebutenolides. The capability to produce natural products with novel chemical scaffolds, such as salimabromide, will ensure the continued investigation of marine myxobacteria as a resource for the discovery of new therapeutics.

## Figures and Tables

**Table 1:** Putative osmolyte synthases and transporters from sequenced marine myxobacteria [22]. BCCT—betaine/carnitine/choline transporter.

Strain	Gene	NCBI Reference Sequence	Length (aa)	Highest Homology	Identity (%)
*H. ochraceum*	*ectC*	WP_012827762.1	142	ectoine synthase (*Hydrogenophaga crassostreae*)	67%
	*betT*	WP_012829907.1	538	BCCT family transporter (*Desulfovermiculus halophilus*)	64%
	*sodium/proline symporter*	WP_012825704.1	598	hypothetical protein (*Hymenobacter terrenus*)	49%
*E. salina*	*ectC*	WP_106088059.1	126	ectoine synthase (*Blastopirellula marina*)	63%
	*ectD*	AMH38938.1	298	ectoine hydroxylase (*Blastopirellula marina*)	59%
	*betT*	AMH38943.1	492	BCCT family transporter (*Spingomonas* sp. Leaf30)	57%
	*sodium/proline symporter*	WP_106088061.1	481	sodium/proline symporter (*Rubinisphaera brasiliensis*)	59%
	*sodium/glutamate symporter*	KIG18073.1	469	hypothetical protein (*P. pacifica*)	64%
*P. pacifica*	*betT* (BCCT family transporter)	EDM75025.1	512	BCCT family transporter (*Spingomonas* sp. Leaf10)	40%
	*sodium/proline symporter*	WP_006976305.1	484	sodium/proline symporter (*E. salina*)	56%
	*sodium/glutamate symporter* (hypothetical protein)	WP_006969752.1	478	sodium/glutamate symporter (*E. salina*)	64%

## References

[1] Reichenbach H. (1999). The ecology of the myxobacteria. Environ. Microbiol..

[2] Stanier R.Y. (1942). A Note on the Elasticotaxis in Myxobacteria. J. Bacteriol..

[3] Stanier R.Y. (1947). Studies on nonfruiting myxobacteria. I. *Cytophaga johnsonae* n. sp., a chitin-decomposing myxobacterium. J. Bacteriol..

[4] Veldkamp H. (1961). A Study on Two Marine Agar-Decomposing, Facultatively Anaerobic Myxobacteria. Microbiology.

[5] Mitchell T.G., Hendrie M.S., Shewan J.M. (1969). The Taxonomy, Differentiation and Identification of *Cytophaga* Species. J. Appl. Bacteriol..

[6] Brockman E.R. (1967). Fruiting myxobacteria from the South Carolina coast. J. Bacteriol..

[7] Rückert G. (1984). Investigations on the distribution of myxobacteria in substrates influenced by seawater with special reference to the island of Helgoland. Helogländer Meeresuntersuchungen.

[8] Gray J.P., Herwig R.P. (1996). Phylogenetic analysis of the bacterial communities in marine sediments. Appl. Environ. Microbiol..

[9] Ravenschlag K., Sahm K., Pernthaler J., Amann R. (1999). High bacterial diversity in permanently cold marine sediments. Appl. Environ. Microbiol..

[10] Bernardet J.F., Segers P., Vancanneyt M., Berthe F., Kersters K., Vandamme P. (1996). Cutting a Gordian Knot: Emended Classification and Description of the Genus *Flavobacterium*, Emended Description of the Family *Flavobacteriaceae*, and Proposal of *Flavobacterium hydatis* nom. nov. (Basonym, *Cytophaga aquatilis* Strohl and Tait 1978). Int. J. Syst. Bacteriol..

[11] Moyer C.L., Dobbs F.C., Karl D.M. (1995). Phylogenetic diversity of the bacterial community from a microbial mat at an active, hydrothermal vent system, Loihi Seamount, Hawaii. Appl. Environ. Microbiol..

[12] Iizuka T., Jojima Y., Fudou R., Yamanaka S. (1998). Isolation of myxobacteria from the marine environment. FEMS Microbiol. Lett..

[13] Zhang Y.Q., Li Y.Z., Wang B., Wu Z.H., Zhang C.Y., Gong X., Qui Z.J., Zhang Y. (2005). Characteristics and living patterns of marine myxobacterial isolates. Appl. Environ. Microbiol..

[14] Li Z.F., Li X., Liu H., Liu X., Han K., Wu Z.H., Hu W., Li F.F., Li Y.Z. (2011). Genome sequence of the halotolerant marine bacterium *Myxococcus fulvus* HW-1. J. Bacteriol..

[15] Horton T., Kroh A., Ahyong S., Bailly N., Boury-Esnault N., Brandão S.N., Costello M.J., Gofas S., Hernandez F., Mees J. (2018). World Register of Marine Species.

[16] Pye C.R., Bertin M.J., Lokey R.S., Gerwick W.H., Linington R.G. (2017). Retrospective analysis of natural products provides insights for future discovery trends. Proc. Natl. Acad. Sci. USA.

[17] Herrmann J., Fayad A.A., Müller R. (2017). Natural Products from myxobacteria: Novel metabolites and bioactivities. Nat. Prod. Rep..

[18] Dávila-Céspedes A., Hufendiek P., Crüsemann M., Schäberle T.F., König G.M. (2016). Marine-derived myxobacteria of the suborder Nannocystineae: An underexplored source of structurally intriguing and biologically active metabolites. Beilstein J. Org. Chem..

[19] Schäberle T.F., Lohr F., Schmitz A., König G.M. (2014). Antibiotics from myxobacteria. Nat. Prod. Rep..

[20] Da Costa M.S., Santos H., Galinski E.A. (1998). An overview of the role and diversity of compatible solute in Bacteria and Archaea. Adv. Biochem. Eng. Biotechnol..

[21] Burg M.B., Ferraris J.D. (2008). Intracellular organic osmolytes: Function and regulation. J. Biol. Chem..

[22] Moghaddam J.A., Boehringer N., Burdziak A., Kunte H., Galinski E.A., Schäberle T.F. (2016). Different strategies of osmoadaptation in the closely related marine myxobacteria *Enhygromyxa salina* SWB007 and *Plesiocystis pacifica* SIR-1. Microbiology.

[23] Schumacher D., Søgaard-Anderson L. (2017). Regulation of Cell Polarity in Motility and Cell Division in *Myxococcus xanthus*. Annu. Rev. Microbiol..

[24] Muñoz-Dorado J., Marcos-Torres F.J., Garćia-Bravo E., Moraleda-Muñoz A., Pérez J. (2016). Myxobacteria: Moving, Killing, Feeding, and Surviving Together. Front. Microbiol..

[25] Wang B., Hu W., Liu H., Zhang C.Y., Zhao J.Y., Jiang D.M., Wu Z.H., Li Y.Z. (2007). Adaptation of Salt-tolerant Myxococcus Strains and their Motility Systems to the Ocean Conditions. Microb. Ecol..

[26] Iizuka T., Jojima Y., Hayakawa A., Fujii T., Yamanaka S., Fudou R. (2013). *Pseudenhygromyxa salsuginis* gen. nov., sp. nov., a myxobacterium isolated from an estuarine marsh. Int. J. Syst. Evol. Microbiol..

[27] Iizuka T., Jojima Y., Fudou R., Tokura M., Hiraishi A., Yamanaka S. (2003). *Enhygromyxa salina* gen. nov., sp. nov., a slightly halophilic myxobacterium isolated from the coastal areas of Japan. Syst. Appl. Microbiol..

[28] Iizuka T., Jojima Y., Fudou R., Hiraishi A., Ahn J.W., Yamanaka S. (2003). *Plesiocystis pacifica* gen. nov., sp. nov., a marine myxobacterium that contains dehydrogenated menaquinone, isolated from the Pacific coasts of Japan. Int. J. Syst. Evol. Microbiol..

[29] Fudou R., Jojima Y., Iizuka T., Yamanaka S. (2002). *Haliangium ochraceum* gen. nov., sp. nov. and *Haliangium tepidum* sp. nov.: Novel moderately halophilic myxobacteria isolated from coastal saline environments. J. Gen. Appl. Microbiol..

[30] Schäberle T.F., Goralski E., Neu E., Erol O., Hölzl G., Dörmann P., Bierbaum G., König G.M. (2010). Marine myxobacteria as a source of antibiotics—Comparison of physiology, polyketide-type genes and antibiotic production of three new isolates of *Enhygromyxa salina*. Mar. Drugs.

[31] Brinkhoff T., Fischer D., Vollmers J., Voget S., Beardsley C., Thole S., Mussmann M., Kunze B., Wagner-Döbler I., Daniel R. (2012). Biogeography and phylogenetic diversity of a cluster of exclusively marine myxobacteria. ISME J..

[32] Jiang D.M., Kato C., Zhou X.W., Wu Z.H., Sato T., Li Y.Z. (2010). Phylogeographic separation of marine and soil myxobacteria at high levels of classification. ISME J..

[33] Kumar S., Stecher G., Tamura K. (2016). MEGA7: Molecular Evolutionary Genetics Analysis version 7.0 for bigger datasets. Mol. Biol. Evol..

[34] Ivanova N., Daum C., Lang E., Abt B., Kopitz M., Saunders E., Lapidus A., Lucas S., Glavina Del Rio T., Nolan M. (2010). Complete genome sequence of *Haliangium ochraceum* type strain (SMP-2). Stand. Genom. Sci..

[35] Fudou R., Iizuka T., Yamanaka S. (2001). Haliangicin, a novel antifungal metabolite produced by a marine myxobacterium. 1. Fermentation and biological characteristics. J. Antibiot. (Tokyo).

[36] Fudou R., Iizuka T., Sato S., Ando T., Shimba N., Yamanaka S. (2001). Haliangicin, a novel antifungal metabolite produced by a marine myxobacterium. 2. Isolation and structural elucidation. J. Antibiot. (Tokyo).

[37] Kundim B.A., Itou Y., Sakagami Y., Fudou R., Iizuka T., Yamanaka S., Ojika M. (2003). New haliangicin isomers, potent antifungal metabolites produced by a marine myxobacterium. J. Antibiot. (Tokyo).

[38] Sun Y., Feng Z., Tomura T., Suzuki A., Miyano S., Tsuge T., Mori H., Suh J.-W., Iizuka T., Fudou R. (2016). Isolation and Biosynthetic Analysis of Haliamide, a New PKS-NRPS Hybrid Metabolite from the Marine Myxobacterium Haliangium ochraceum. Molecules.

[39] Sun Y., Feng Z., Tomura T., Suzuki A., Miyano S., Tsuge T., Mori H., Suh J., Iizuka T., Fudou R. (2016). Heterologous Production of the Marine Myxobacterial Antibiotic Haliangicin and Its Unnatural Analogues Generated by Engineering of the Biochemical Pathway. Sci. Rep..

[40] Timmermans M.L., Paudel Y.P., Ross A.C. (2017). Investigating the Biosynthesis of Natural Products from Marine Proteobacteria: A Survey of Molecules and Strategies. Mar. Drugs.

[41] Amiri Moghaddam J., Poehlein A., Fisch K., Alanjary M., Daniel R., König G.M., Schäberle T.F. (2018). Draft Genome Sequences of the Obligatory Marine Myxobacterial Strains *Enhygromyxa salina* SWB005 and SWB007. Genome Announc..

[42] Garcia R., Gerth K., Stadler M., Dogma I.J., Müller R. (2010). Expanded phylogeny of myxobacteria and evidence for cultivation of the ‘unculturables’. Mol. Phylogenet. Evol..

[43] Felder S., Felder S., Dreisigacker S., Kehraus S., Neu E., Bierbaum G., Wright P.R., Menche D., Schäberle T.F., König G.M. (2013). Salimabromide: Unexpected chemistry from the obligate marine myxobacterium *Enhygromyxa salina*. Chemistry.

[44] Felder S., Kehraus S., Neu E., Bierbaum G., Schäberle T.F., König G.M. (2013). Salimyxins and enhygrolides: Antibiotic, sponge-related metabolites from the obligate marine myxobacterium *Enhygromyxa salina*. ChemBioChem.

[45] Blin K., Wolf T., Chevrette M.G., Lu X., Schwalen C.J., Kautsar S.A., Suarez Duran H.G., de Los Santos E.L.C., Kim H.U., Nave M. (2017). antiSMASH 4.0-improvements in chemistry prediction and gene cluster boundary identification. Nucleic Acids Res..

[46] Komaki H., Fudou R., Iizuka T., Nakajima D., Okazaki K., Shibata D., Ojika M., Harayama S. (2008). PCR detection of type I polyketide synthase genes in myxobacteria. Appl. Environ. Microbiol..

[47] Bogdanović X., Hesseler M., Palm G.J., Bornscheuer U.T., Hinrichs W. (2010). Crystallization and preliminary X-ray diffraction studies of the putative haloalkane dehalogenase DppA from *Plesiocystis pacifica* SIR-1. Acta Crystallogr. Sect. F Struct. Biol. Cryst. Commun..

[48] Hesseler M., Bogdanović X., Hidalgo A., Berenguer J., Palm G.J., Hinrichs W., Bornscheuer U.T. (2011). Cloning, functional expression, biochemical characterization, and structural analysis of a haloalkane dehalogenase from *Plesiocystis pacifica* SIR-1. Appl. Microbiol. Biotechnol..

[49] Tomura T., Nagashima S., Yamazaki S., Iizuka T., Fudou R., Ojika M. (2017). An Unusual Diterpene-Enhygromic Acid and Deoxyenhygrolides from a Marine Myxobacterium, *Enhygromyxa* sp.. Mar. Drugs.

[50] Ojika M., Inukai Y., Kito Y., Hirata M., Iizuka T., Fudou R. (2008). Miuraenamides: Antimicrobial cyclic depsipeptides isolated from a rare and slightly halophilic myxobacterium. Chem. Asian J..

[51] Iizuka T., Fudou R., Jojima Y., Ogawa S., Yamanaka S., Inukai Y., Ojika M. (2006). Miuraenamides A and B, Novel Antimicrobial Cyclic Depsipeptides from a New Slightly Halophilic Myxobacterium: Taxonomy, Production, and Biological Properties. J. Antibiot..

